# Associations of corticosteroid therapy with weight change and appetite in patients with advanced pancreatic cancer – a post hoc analysis from the MISTRAL trial

**DOI:** 10.1007/s00520-026-10585-2

**Published:** 2026-03-28

**Authors:** Charlotte Goodrose-Flores, Stephanie E. Bonn, Linda Björkhem-Bergman, Kathrin Wode

**Affiliations:** 1 Division of Clinical Geriatrics, Department of Neurobiology, Care Sciences and Society (Huddinge), Karolinska Institutet, 141 83 Huddinge, Sweden; 2 Division of Clinical Epidemiology, Department of Medicine (Solna), Karolinska Institutet, Huddinge, Sweden; 3 Palliative Medicine and R&D Unit, Stockholms Sjukhem, Mariebergsgatan 22, 112 19 Stockholm, Sweden

**Keywords:** Appetite, Corticosteroid therapy, Weight

## Abstract

**Background:**

Loss of appetite and weight loss are major concerns in patients with pancreatic cancer. The aim of this study was to evaluate the associations of corticosteroid therapy with weight change and appetite in the long term, in patients with advanced pancreatic cancer.

**Methods:**

This was a post hoc analysis of the previously performed randomized, controlled MISTRAL trial in patients with advanced pancreatic cancer. Data on weight and appetite at baseline and after approximately 1, 2, 3 and 4 months were used. The association between corticosteroid exposure and weight change was calculated using mixed linear regression, models adjusted for sex, age, randomization arm and performance status. Appetite was analyzed comparing those without and with corticosteroids during 3 days before appetite assessment.

**Results:**

Two hundred forty-four patients (121 women) were included. Patients who received corticosteroid therapy at any time during the 4 months had less appetite (*p* < 0.001), lower performance status (*p* < 0.001) and lower BMI (*p* < 0.05) compared to those not receiving corticosteroids. Patients receiving corticosteroids had a larger weight loss of on average 1.48% during a month of follow-up compared to those not receiving corticosteroids (*β* − 1.48, 95% CI =  − 2.11 to − 0.84). Patients exposed to corticosteroids reported poorer appetite at every time point than those not exposed.

**Conclusion:**

In long-term follow-up, corticosteroid treatment was associated with greater weight loss and poorer appetite among patients with advanced pancreatic cancer. These findings may partly be explained by a higher symptom burden among patients receiving corticosteroids. Future prospective trials are warranted to clarify the effect of long-term corticosteroid treatment on weight and appetite.

## Background

Loss of appetite is a major concern in patients with advanced cancer [1]. A multimodal approach is suggested to mitigate potential weight loss, including assuring adequate energy intake, as it might slow down weight loss and prolong life [2–4]. Additionally, pancreas cancer–associated weight loss contributes to a decline in quality of life and an increase in overall mortality [5–7], highlighting the importance of interventions. Weight loss should be identified and addressed early in the progression of the disease [3, 8].

Cancer tumors increase resting energy expenditure, causing hypermetabolism and thereby increasing the energy requirements of the patient. This appears in response to increased levels of TNF-α, CRP, IL-1, IL-6, GDF-15, among others, as well as ghrelin resistance. Further, more than half of patients diagnosed with pancreatic cancer develop liver metastases [9] which also increase energy expenditure. The energy requirement of a healthy liver is approximately 200 kcal per day, but can increase by an additional 343 kcal per day in the presence of liver metastases [10].

The altered metabolism challenges the patient’s ability to maintain energy balance and meet increased protein needs [3, 11]. Malnutrition is a serious consequence that may lead to loss of muscle mass and subcutaneous fat, as well as a decline in functional status [2, 12]. It has been established that maintaining body mass index (BMI) during the disease, regardless of the BMI pre-diagnosis, decreases mortality [13]. Therefore, interventions to improve appetite are a priority in clinical cancer care [14].

Corticosteroid therapy is extensively used in cancer care throughout the whole disease trajectory—both in curative and palliative care. One of the indications for corticosteroid treatment is to improve appetite in patients with advanced cancer [15, 16]. However, corticosteroid therapy increases the risk of infection and proximal myopathy, and therefore cannot be recommended as standard care [16, 17]. The side effects of corticosteroid therapy, such as weight gain and increased appetite, are in other patient populations, often considered unwanted consequences [18, 19].

Significant knowledge gaps remain regarding the effects of corticosteroid therapy on appetite and weight change in advanced cancer overall, and in pancreatic cancer specifically.

Mistletoe extract administered subcutaneously is recognized as complementary cancer treatment and the effect of mistletoe on overall survival in patients with advanced pancreatic cancer was evaluated in the randomized, placebo-controlled, double-blind MISTRAL trial [20]. The trial showed no significant benefit of adding mistletoe to standard treatment in terms of overall survival, health-related quality of life, corticosteroid therapy or body weight [20, 21]. Hence, there was no significant difference between the two groups.

The primary aim of this post hoc study was to address the association between corticosteroid therapy and weight change in patients with advanced pancreatic cancer. Secondly, we also aimed to address the association between corticosteroid therapy and appetite. To this end, we used data from the MISTRAL trial.

## Methods

We have performed post hoc analyses using data from the MISTRAL trial, EU Clinical Trial Register, EudraCT Number 2014–004552-64, and registration NCT02948309, on October 28, 2016, a Swedish multicentred, parallel group, double-blind, randomized, placebo-controlled clinical trial. The trial has been described in detail elsewhere [20, 22]. In brief, 290 patients with advanced pancreatic cancer were recruited and randomized 1:1 to either mistletoe extract or placebo in addition to standard treatment (palliative chemotherapy or best supportive care) during June 2016 to December 2021. All participants had access to symptom management and to palliative care (specialized multidisciplinary home or inpatient care) if needed. Drugs for symptom relief (e.g. analgesics, pancreatic enzyme therapy (PET)) were registered for indication, dose and date; antiemetic drugs within the context of palliative chemotherapy were part of national standardized protocols.

**Fig. 1:**
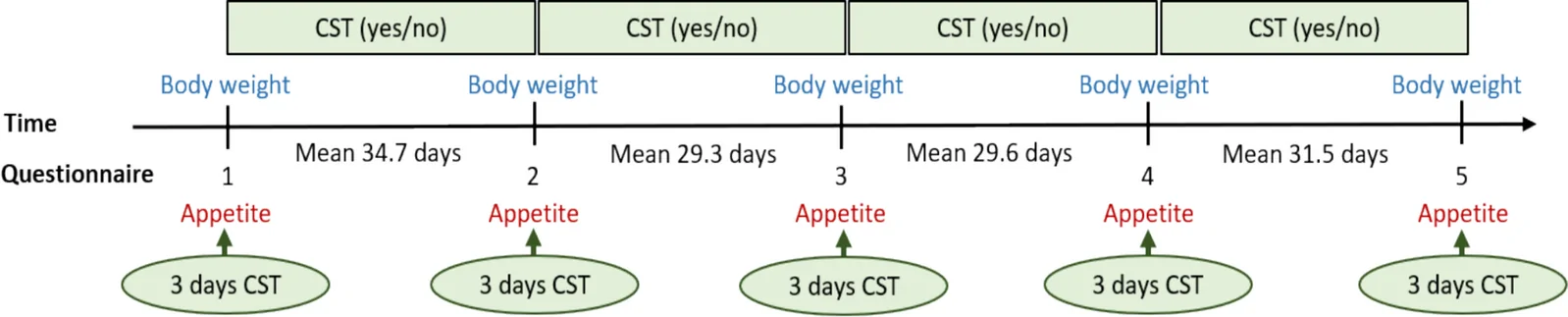
Outline of corticosteroid therapy (CST) exposure assessment in relation to weight measurements and appetite assessments in the MISTRAL trial

Main inclusion criteria included a recent diagnosis of advanced exocrine pancreatic cancer or relapse, an Eastern Cooperative Oncology Group (ECOG) performance status of ≤ 2, and a life expectancy of more than 4 weeks. Study assessments were performed at study visits at the time of randomization (baseline) and after 5 to 6 weeks, and at 2, 3, 4, 6 and 9 months after randomization. On each study visit, participants responded to a HRQoL questionnaire (EORTC QLQ-C30 and EORTC PAN-26) [23, 24], including assessment of appetite. The present study has analyzed data from the first five study assessments, i.e. the first 4 months of follow-up, in the MISTRAL trial. The timespan was chosen due to similar follow-up times of approximately 4 weeks between assessments. Figure 1 outlines the exposures (corticosteroid therapy) and outcomes (weight and appetite) used in the present study.

### Study population

**Fig. 2:**
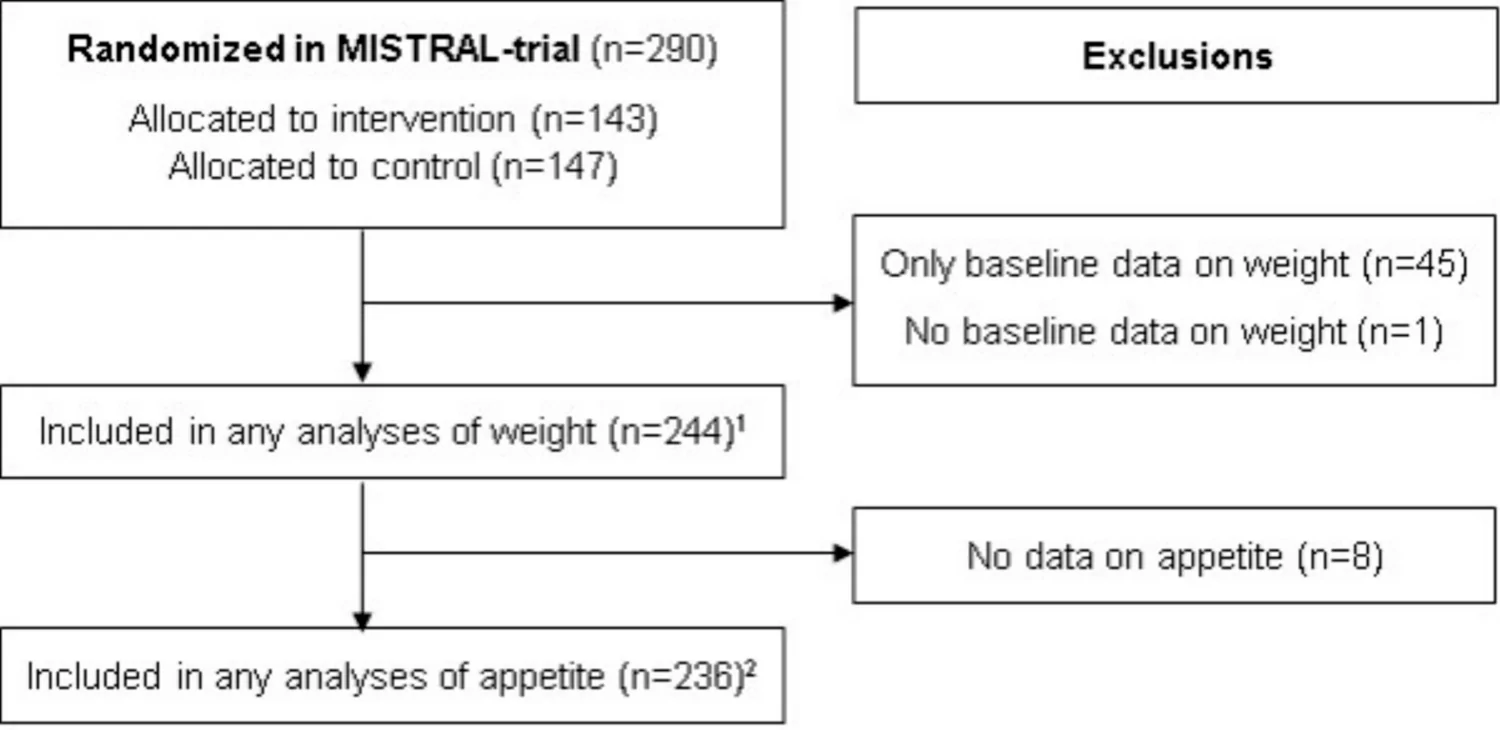
Flow-chart of participants in the present study. A flow chart outlining participants in the full MISTRAL trial can be found elsewhere [20, 22]. ^1^Data on weight at baseline and at least one follow-up; ^2^Data on appetite from at least one questionnaire during follow-up

In this study addressing weight change over time, MISTRAL participants that only had baseline data on weight (*n* = 45) or with missing body weight at baseline (*n* = 1) were excluded. The primary analytical dataset comprised 244 men and women, thus representing a subset of the original MISTRAL population. In analyses of appetite, participants with no data on appetite from any of the five study questionnaires during the first 4 months (*n* = 8) were further excluded. All included participants had data on weight at baseline and from at least one additional follow-up, and appetite data from at least one questionnaire, but not necessarily from the baseline questionnaire. Therefore, the number of participants included in the analyses varies depending on the type of analysis. A flow chart of the study participants in the present study is shown in Fig. 2.

### Exposure variables—corticosteroid therapy

To address the association between corticosteroid exposure and weight change, variables defining corticosteroid exposure (yes/no) between study questionnaires were created. Most participants in our data with corticosteroid therapy received betamethasone (98.3%) representing the most common used corticosteroid during chemotherapy and for symptom relief in Sweden. Corticosteroid treatment as an antiemetic drug for palliative chemotherapy followed national standard antiemetic protocols and ranged from 4 mg for low emetogenic chemotherapy (e.g. gemcitabine) up to 36 mg per cycle for high emetogenic treatments (e.g. FOLFIRINOX). This corticosteroid use was not included in our analysis since cytostatic treatment is likely to have an extensive impact on the patient’s appetite, potentially outweighing the appetite-stimulating effects of corticosteroids.

Four variables corresponding to the time intervals between study questionnaires one and two, two and three, three and four, and four and five were created. A variable defining corticosteroid exposure during the first 4 months of follow-up, i.e. between the first questionnaire and the fifth questionnaire, was also created. For each time period, days with a prescribed treatment were divided by the total number of days during the period.

To address the direct association between corticosteroid therapy and appetite, variables defining corticosteroid therapy exposure occurring simultaneously with reported appetite in the five study questionnaires were derived. Participants were considered exposed to corticosteroid therapy if they used corticosteroids during all 3 days prior to the date of responding to each respective study questionnaire. Participants with ongoing palliative chemotherapy during any one of the 3 days used to define corticosteroid exposure were excluded from this analysis since the effect on appetite from the palliative chemotherapy would potentially outweigh the effect of corticosteroids. This could occur if a participant had been prescribed corticosteroid therapy for another reason than palliative chemotherapy and received it as part of palliative chemotherapy.

### Outcome variables—body weight

Body weight was measured at home weekly and recorded in kilograms with one decimal in a study-specific patient diary. Weight was also measured at the clinic at every study visit. For the present study, measured weights from diaries recorded within 14 days of the questionnaire date were primarily used. Secondly, if the participant did not have a recorded weight within this time frame, measured weights at study visits were used given that the measurement fell within the limit of 14 days from responding to the questionnaire. If a participant had more than one weight recording within the given time frame, the weight recorded closest to the questionnaire date was used. In this study, 78.5% of participants had only diary-recorded weights included in the exposure. Concordance between self-reported and measured weights was assessed by comparing clinic weights from the first study visit with self-reported diary weights recorded within ± 5 days. This time window was chosen to maximize the number of participants with both measurements (*n* = 136) while minimizing the likelihood of true changes in body weight. Self-reported and clinic-measured weights were highly correlated (Pearson’s *r* = 0.97, *p* < 0.001), with a mean difference of 0.5 kg.

### Outcome variables—appetite

Appetite was assessed in each study questionnaire using the EORTC QLQ-C30 [23]. Study participants responded to the question “Have you lacked appetite?” on a 4-point Likert scale with response alternatives 1 (“Not at all”), 2 (“A little”), 3 (“Quite a bit”) and 4 (“Very much”). A dichotomous variable defining appetite at each study visit was also created by combining response alternatives “Not at all”and “A little”, indicating good appetite, and response alternatives “Quite a bit” and “Very much”, indicating poor appetite.

### Other variables

Information on age and sex at baseline was derived from medical journals. Time from diagnosis to randomization was calculated from the date of primary diagnosis of pancreatic cancer or a relapse of the previous pancreatic cancer. Survival time was calculated from the time from randomization to death of any cause or truncated at the end of the MISTRAL trial period. Participants’ functional status was assessed at each study visit using the ECOG scale running from 0 (fully active) to 4 (completely disabled) [25]. The ECOG scale assesses functional status in terms of ability to care for oneself, perform daily activities and perform physical activities, e.g. walking or working. To assess an eventual influence of PET on steroid use, we analyzed PET use in participants with and without corticosteroid therapy. Existing comorbidities within our study population were categorized with the five largest groups comprising > 15 participants being diseases of the circulatory system (ICD 10 I), endocrine diseases (ICD 10 E), respiratory diseases (ICD 10 J), diseases of the musculoskeletal system and connective tissue (ICD 10 M) and diseases of the digestive system (ICD 10 K). Other comorbidities were present but occurred in only a few participants.

### Statistical analyses

Characteristics of study participants are presented for all participants and by sex using mean values with standard deviations (SD) for continuous variables and numbers and percentages for categorical variables. Age at baseline, and weight, BMI, sex distribution, ECOG status and appetite at the beginning and end (weight and BMI) of each study time interval between questionnaires are presented among participants with or without corticosteroid exposure within each interval. Difference between sexes and between participants with or without corticosteroid exposure was analyzed using independent group *t*-tests for continuous variables and chi-2 tests for categorical variables. The association between corticosteroid exposure during the four different time intervals between study assessments, and percentage of weight change during the same interval was analyzed using linear regression for each separate interval. Only participants with weight at both the beginning and end of an interval are included in the analyses of that specific time period. An overall analysis including data from all intervals was modelled using linear mixed model regression with random intercept and slope for each participant over time. The exposure was modelled as a categorical variable with levels of no corticosteroid exposure (reference level) and any percentage of corticosteroid exposure. Models were adjusted for age, sex, and study group, and ECOG status and weight at the starting time point of each interval. Resulting beta-coefficients are interpreted as the difference in weight change during a month between exposure groups. To address the association and weight change over a longer period, we also performed analyses of corticosteroid exposure during the whole 4-month follow-up and weight change during the same period. The model was adjusted for age, sex, study group, ECOG status and weight at baseline. All models were also run stratified by sex. To account for potential confounding introduced by sicker participants passing away during follow-up and illness also being related to exposure to corticosteroid therapy, sensitivity analyses only including participants that completed the study questionnaire after 4 months of follow-up (*n* = 174) were performed.

Differences in appetite among those with a 3-day corticosteroid exposure before each appetite assessment were tested with chi-2 tests. Further, appetite after a period of no corticosteroid exposure was compared to appetite after a period of corticosteroid exposure within individuals. Only individuals that had the combination of no corticosteroid exposure before an appetite assessment followed by corticosteroid exposure before the next appetite assessment were included in analyses (*n* = 46, 28 men and 18 women). In these analyses, a dichotomous variable of lack of appetite (no/yes) was created. Difference in appetite between corticosteroid exposure/no exposure within individuals was tested using McNemar’s test for paired dichotomous data.

All analyses were performed using STATA 17.0, with the level of statistical significance set at *p* < 0.05.

## Results

Baseline characteristics of participants included in the current analyses are presented for all, and by sex, in Table 1. Among the 244 participants, 121 were women. The mean age and BMI at baseline were 68.0 years and 23.7 kg/m^2^, respectively. Most participants reported no (38.0%) or a slight decrease in appetite (28.2%) at baseline, and had an ECOG status of 0 or 1 (85.8%). Most participants received palliative chemotherapy (84.8%). There were no statistically significant differences in baseline characteristics between men and women. The median survival time from inclusion was 9.0 months in the current study cohort. In total, 184 of the 244 participants (75.4%) used PET. PET use was slightly more common in women (96/121, 79.3%) than men (88/123, 71.5%); however, this difference was not statistically significant (*p* = 0.16). The proportion of participants with diseases of the circulatory system was significantly higher in men (58.5%) than in women (41.3%, *p* = 0.007). No other significant sex differences were observed (data not shown).

**Table 1:** Baseline characteristics of study participants in the current study cohort, a post-hoc analysis from the MISTRAL trial

	All (*n* = 244)		Men (*n* = 123)		Women (*n* = 121)		*p*^1^
	**Mean**	**(SD)**	**Mean**	**(SD)**	**Mean**	**(SD)**	
Age, years	68.0	(9.7)	68.6	(10.2)	67.3	(9.2)	0.30
Weight, kg	70.4	(14.7)	76.2	(11.8)	64.5	(14.9)	< 0.001
BMI^2^, kg/m^2^	23.7	(4.3)	24.1	(3.6)	23.4	(4.8)	0.17
	**Median**	**(IQR)**	**Median**	**(IQR)**	**Median**	**(IQR)**	0.65
Time from diagnosis to randomization, days	41	(28–64)	43	(28–64)	36	(27–64)	
	*n*	(%)	*n*	(%)	*n*	(%)	
Intervention group							0.90
Mistletoe	122	(50.0)	61	(49.6)	61	(50.4)	
Control	122	(50.0)	62	(50.4)	60	(49.6)	
Lack of appetite^3^							0.99
Not at all	89	(38.0)	46	(39.0)	43	(37.1)	
A little	66	(28.2)	33	(28.0)	33	(28.5)	
Quite a bit	47	(20.1)	23	(19.5)	24	(20.7)	
Very much	32	(13.7)	16	(13.6)	16	(13.8)	
ECOG performance status at baseline							0.44
0	115	(47.1)	53	(43.1)	62	(51.2)	
1	94	(38.5)	51	(41.5)	43	(35.5)	
2	35	(14.3)	19	(15.5)	16	(13.2)	
Received standard treatment							0.056
Palliative chemotherapy	207	(84.8)	99	(80.5)	108	(89.3)	
Best supportive care (BSP)	37	(15.2)	24	(19.5)	13	(10.7)	

^1^Independent group *t*-test for continuous variables and chi-2 test for categorical variables comparing means and distribution, respectively, between sexes. ^2^Missing data on height (*n* = 1). ^3^Missing data on appetite in baseline questionnaire (*n* = 10)

Corticosteroids were primarily prescribed for improvement of general condition, to improve appetite, or to manage pain or nausea. The prescribed corticosteroids doses ranged between 0.5 and 12 mg betamethasone/day. Results comparing characteristics, ECOG performance status and appetite between participants with or without corticosteroid therapy during follow-up are presented in Table 2. There were no statistically significant differences in age, weight or sex distribution between those that were exposed to corticosteroid therapy or not during the different follow-up time periods. Participants with corticosteroid therapy exposure reported significantly lower BMI at the end of all time periods (*p* < 0.05), except the first (*p* = 0.057). In the following time periods, those exposed to corticosteroid therapy also had a statistically significantly lower BMI at the start of each period. Participants who received corticosteroid therapy had a lower ECOG performance status and reduced appetite at all time points compared to those who did not (*p* < 0.001). Additionally, there was no difference in the proportion of PET users between participants with corticosteroid therapy (71.4%–82.4%) and those without corticosteroid therapy (75.5%–76.9%) across any of the four time periods defined between questionnaires (*p* = 0.40–0.60). There were no statistically significant differences in the prevalence of any of the five comorbidity categories between participants with and without corticosteroid exposure across time periods (*p* = 0.09–0.94; data not shown).

**Table 2:** Comparison of baseline characteristics between participants with or without corticosteroid exposure during the four time periods between questionnaires and for the full time including all four time periods

		Corticosteroid exposure				*p*^1^
Time period 1		**No** (*n* = 189)		**Yes** (*n* = 55)		
		**Mean**	**(SD)**	**Mean**	**(SD)**	
Age at baseline, years		67.4	(9.7)	69.9	(9.6)	0.09
Weight at start of period, kg		70.7	(15.7)	69.3	(10.4)	0.53
Weight at end of period, kg		70.0	(15.5)	68.3	(10.3)	0.44
BMI at start of period, kg/m^2^		24.0	(4.5)	22.9	(3.1)	0.09
BMI at end of period, kg/m^2^		23.8	(4.4)	22.5	(3.2)	0.057
		*n*	(%)	*n*	(%)	
Sex	Men	93	(49.2)	30	(54.6)	0.49
	Women	96	(50.8)	25	(45.5)	
ECOG performance status at start of period^2^	0	97	(51.3)	18	(32.7)	**0.001**
	1	73	(38.6)	21	(38.2)	
	2	19	(10.1)	16	(29.1)	
	3	0	-	0	-	
	4	0	-	0	-	
Lack of appetite at start of period	Not at all	80	(43.7)	9	(17.7)	** < 0.0001**
	A little	54	(29.5)	12	(23.5)	
	Quite a bit	33	(18.0)	14	(27.5)	
	Very much	16	(8.7)	16	(31.4)	
	Missing data	6		4		
Time period 2		**No** (*n* = 169)		**Yes** (*n* = 49)		
		**Mean**	**(SD)**	**Mean**	**(SD)**	
Age at baseline, years		67.9	(9.1)	68.1	(10.9)	0.89
Weight at start of period, kg		70.1	(15.7)	69.7	(11.1)	0.87
Weight at end of period, kg		70.3	(15.7)	68.0	(10.5)	0.35
BMI at start of period, kg/m^2^		23.8	(4.4)	22.8	(3.4)	0.15
BMI at end of period, kg/m^2^		23.9	(4.4)	22.4	(3.1)	**0.03**
		*n*	(%)	*n*	(%)	
Sex	Men	80	(47.3)	26	(53.1)	0.48
ECOG performance status at start of period	Women	89	(52.7)	23	(46.9)	
	0	83	(49.7)	8	(16.7)	** < 0.0001**
	1	62	(37.1)	23	(47.9)	
	2	21	(12.6)	12	(25.0)	
	3	1	(0.6)	5	(10.4)	
	4	0	-	0	-	
	Missing data	2		1		
Lack of appetite at start of period	Not at all	87	(51.8)	11	(23.4)	** < 0.0001**
	A little	47	(28.0)	12	(25.5)	
	Quite a bit	26	(15.5)	10	(21.3)	
	Very much	8	(4.8)	14	(29.8)	
	Missing data	1		2		
Time period 3		**No** (*n* = 151)		**Yes** (*n* = 45)		
		**Mean**	**(SD)**	**Mean**	**(SD)**	
Age at baseline, years		67.8	(9.1)	68.5	(11.0)	0.70
Weight at start of period, kg		70.2	(15.9)	69.6	(10.9)	0.81
Weight at end of period, kg		70.2	(15.9)	69.2	(10.3)	0.69
BMI at start of period, kg/m^2^		24.0	(4.5)	22.7	(3.0)	0.079
BMI at end of period, kg/m^2^		24.0	(4.6)	22.4	(2.9)	**0.037**
		*n*	(%)	*n*	(%)	
Sex	Men	68	(45.0)	27	(60.0)	0.078
ECOG performance status at start of period	Women	83	(55.0)	18	(40.0)	
	0	74	(51.8)	7	(15.6)	** < 0.0001**
	1	55	(38.5)	19	(42.2)	
	2	14	(9.8)	14	(31.1)	
	3	0	-	5	(11.1)	
	4	0	-	0	-	
Lack of appetite at start of period	Missing data	8		0		
	Not at all	76	(51.0)	12	(27.9)	**0.001**
	A little	50	(33.6)	14	(32.6)	
	Quite a bit	19	(12.8)	10	(23.3)	
	Very much	4	(2.7)	7	(16.3)	
	Missing data	2		2		
Time period 4		**No** (*n* = 143)		**Yes** (*n* = 34)		
		**Mean**	**(SD)**	**Mean**	**(SD)**	
Age at baseline, years		67.7	(8.7)	68.0	(11.4)	0.85
Weight at start of period, kg		70.5	(15.8)	67.2	(11.1)	0.25
Weight at end of period, kg		70.9	(15.8)	67.0	(12.3)	0.19
BMI at start of period, kg/m^2^		24.1	(4.5)	22.1	(3.1)	**0.016**
BMI at end of period, kg/m^2^		24.2	(4.5)	22.0	(3.5)	**0.009**
		*n*	(%)	*n*	(%)	
Sex	Men	66	(46.2)	18	(52.9)	0.48
	Women	77	(53.9)	16	(47.1)	
ECOG performance status at start of period	0	64	(47.1)	8	(24.2)	** < 0.0001**
	1	61	(44.9)	11	(33.3)	
	2	10	(7.4)	11	(33.3)	
	3	1	(0.7)	3	(9.1)	
	4	0	-	0	-	
	Missing data	7		1		
Lack of appetite at start of period	Not at all	96	(68.6)	12	(36.4)	**0.001**
	A little	30	(21.4)	10	(30.3)	
	Quite a bit	9	(6.4)	7	(21.2)	
	Very much	5	(3.6)	4	(12.1)	
	Missing data	3		1		
Time period 1 to 4		**No** (*n* = 130)		**Yes** (*n* = 48)		
		**Mean**	**(SD)**	**Mean**	**(SD)**	
Age at baseline, years		67.6	(9.1)	68.3	(9.9)	0.65
Weight at start of period, kg		70.7	(16.5)	70.0	(11.9)	0.78
Weight at end of period, kg		70.9	(16.3)	68.2	(11.7)	0.30
BMI at start of period, kg/m^2^		24.3	(4.7)	23.0	(3.5)	0.09
BMI at end of period, kg/m^2^		24.3	(4.6)	22.4	(3.4)	**0.009**
		*n*	(%)	*n*	(%)	
Sex	Men	59	(45.4)	26	(54.2)	0.30
	Women	71	(54.6)	22	(45.8)	
ECOG performance status at start of period	0	57	(45.2)	9	(20.0)	** < 0.0001**
	1	57	(45.2)	18	(40.0)	
	2	9	(7.1)	10	(22.2)	
	3	2	(1.6)	7	(15.6)	
	4	1	(0.8)	1	(2.2)	
	Missing data	4		3		
Lack of appetite at start of period	Not at all	80	(63.5)	17	(39.5)	** < 0.0001**
	A little	33	(26.2)	11	(25.6)	
	Quite a bit	10	(7.9)	7	(16.3)	
	Very much	3	(2.4)	8	(18.6)	
	Missing data	4		5		

^1^*T*-test for continuous variables and chi-2 test for categorical variables comparing means and distribution, respectively, between exposure groups. ^2^Inclusion criteria for MISTRAL trial ECOG 0–2. The amount of missing data ranged from *n* = 2 to *n* = 5 for variables of weight and from *n* = 3 to *n* = 6 for BMI

Results from linear regression analysis of the association between corticosteroid exposure and percentage of weight change during the different time intervals are shown in Table 3. Results from the linear mixed models taking all time periods into account within the same model show that being exposed to corticosteroid therapy was associated with a 1.48% larger decrease in body weight (*β* =  − 1.48, 95% CI =  − 2.11 to − 0.84) during a month compared to not being exposed. When stratified by sex, men exposed to corticosteroid therapy had decreased their weight by 1.07% (*β* =  − 1.07, 95% CI =  − 1.91 to − 0.24) compared to unexposed men, while women exposed to corticosteroids had decreased their body weight by 2.02% (*β* =  − 2.02, 95% CI =  − 3.02 to − 1.01) compared to unexposed women. Confidence intervals were largely overlapping between men and women and no sex differences were observed. Results maintained similar in sensitivity analysis only including participants that had completed the study questionnaire after four months of follow-up.

**Table 3:** Results from linear regression models of the association between corticosteroid exposure (no/yes) and percentage of weight change during follow-up among all participants and stratified by sex

Main analyses				Sensitivity analyses^1^		
All						
Time period	*n*	*β*^2^	(95% CI)	*n*	*β*^3^	(95% CI)
1	239	− 1.15	(− 2.42 to 0.12)	174	− 1.49	(− 2.72 to − 0.27)
2	202	− 1.41	(− 2.59 to − 0.22)	170	− 1.03	(− 2.21 to 0.15)
3	177	− 1.35	(− 2.46 to − 0.24)	166	− 1.44	(− 2.66 to − 0.22)
4	163	− 1.81	(− 3.29 to − 0.33)	163	− 1.81	(− 3.29 to − 0.33)
Overall^4^	239	− 1.48	(− 2.11 to − 0.84)	174	− 1.51	(− 2.16 to − 0.86)
Men						
Time period	*n*	*β*^2^	(95% CI)	*n*	*β*^3^	(95% CI)
1	121	− 0.58	(− 1.93 to 0.77)	83	− 1.01	(− 2.81 to 0.79)
2	99	− 1.30	(− 3.10 to 0.51)	81	− 1.90	(− 3.61 to − 0.18)
3	88	− 1.88	(− 3.24 to − 0.53)	81	− 1.72	(− 3.23 to − 0.20)
4	80	− 0.08	(− 2.17 to 2.00)	80	− 0.08	(− 2.17 to 2.00)
Overall^4^	121	− 1.07	(− 1.91 to − 0.24)	83	− 1.28	(− 2.23 to − 0.33)
Women						
Time period	*n*	*β*^2^	(95% CI)	*n*	*β*^3^	(95% CI)
1	118	− 1.78	(− 4.03 to 0.46)	91	− 1.17	(− 2.90 to 0.56)
2	103	− 1.41	(− 3.04 to 0.22)	89	− 0.13	(− 1.85 to 1.60)
3	89	− 0.67	(− 2.60 to 1.26)	85	− 1.09	(− 3.18 to 1.00)
4	83	− 3.51	(− 5.59 to − 1.42)	83	− 3.51	(− 5.59 to − 1.42)
Overall^4^	118	− 2.02	(− 3.02 to − 1.01)	91	− 1.84	(− 2.78 to 0.91)

^1^Including only participants that completed study questionnaire five after 4 months of follow-up. ^2^Adjusted for age at inclusion (continuous), sex (male/female), study group (intervention/placebo) and ECOG at start of time interval (categories 0 to4). ^3^Adjusted for age at inclusion, study group and ECOG at start of time interval. ^4^Mixed model with random intercept and slope for each participant over time

Table 4 shows appetite assessments depending on direct corticosteroid exposure during the 3 days preceding appetite assessment. At each time point, patients who had not been exposed to corticosteroids reported better appetite, i.e. less frequent lack of appetite, compared to those who had been exposed (*p* < 0.05). McNemar’s test for paired dichotomous data showed no significant difference in appetite (dichotomized variable of good vs. poor appetite) between periods of corticosteroid exposure and no exposure (*p* = 0.81). When stratified by sex, the results remained non-significant for both men (*p* = 0.21) and women (*p* = 0.26).

**Table 4:** Appetite assessments in study questionnaire 1 to 5 by exposure of corticosteroid treatment three days prior to the date of responding to each respective questionnaire

	All	Lack of appetite								*p*-value^1^
		Not at all		A little		Quite a bit		Very much		
	*n*	*n*	(%)	*n*	(%)	*n*	(%)	*n*	(%)	
3 days CST before Questionnaire 1										0.018
No	211	84	(39.8)	62	(29.4)	40	(19.0)	25	(11.9)	
Yes	20	4	(20.0)	3	(15.0)	7	(35.0)	6	(30.0)	
3 days CST before Questionnaire 2										0.001
No	200	97	(48.5)	53	(26.5)	32	(16.0)	18	(9.0)	
Yes	32	9	(28.1)	8	(25.0)	4	(12.5)	11	(34.4)	
3 days CST before Questionnaire 3										< 0.0001
No	169	82	(48.5)	55	(32.5)	25	(14.8)	7	(4.1)	
Yes	32	8	(25.0)	8	(25.0)	6	(18.8)	10	(31.3)	
3 days CST before Questionnaire 4										0.023
No	148	96	(64.9)	32	(21.6)	13	(8.8)	7	(4.7)	
Yes	31	11	(35.5)	11	(35.5)	6	(19.4)	3	(9.7)	
3 days CST before Questionnaire 5										0.003
No	142	87	(61.3)	37	(26.1)	12	(8.5)	6	(4.2)	
Yes	22	8	(36.4)	5	(22.7)	4	(18.2)	5	(22.7)	

^1^Chi-2 test of appetite between participants exposed or not exposed the 3 days before appetite assessment

*CST* corticosteroid therapy

## Discussion

We found that, among patients diagnosed with advanced pancreatic cancer, those who received corticosteroid therapy showed greater weight loss each month over the 4-month observation period compared with those who did not receive corticosteroids, indicating an association, after adjusting for age, sex, treatment arm and ECOG. The results remained consistent when analyzing only patients who completed the entire study period. Furthermore, patients who had not been exposed to corticosteroid therapy reported a better appetite than those who had, based on monthly assessments over 4 months. There were no significant differences between men and women.

Corticosteroids have long been prescribed by physicians in palliative care to alleviate symptom burden, such as fatigue, poor appetite, nausea, prevent cachexia and pain [26–29]. It is listed by the WHO as one of the essential medicines for palliative care [30]. In Sweden, betamethasone is the most commonly used corticosteroid [29], while dexamethasone is the most frequently prescribed corticosteroid in palliative care outside of Sweden, due to its long half-life [27, 31]. Notably, the evidence supporting the use of corticosteroids in palliative care remains limited for most indications [27]. The treatment is associated with challenging side effects, such as hyperglycemia, proximal myopathy, peptic ulcers, psychiatric disturbances and insomnia [27]. Still, according to our clinical experience, corticosteroids are often highly effective in increasing appetite and vitality, and in decreasing symptom burden in patients with advanced cancer—but this is mainly seen in the short-term perspective. Corticosteroid therapy in a long-term perspective is seldom evaluated in advanced cancer care.

In our study population, corticosteroids were more frequently prescribed to patients with advanced pancreatic cancer who exhibited loss of appetite, reduced performance status and likely a poorer prognosis. We have not been able to address the causal effect of corticosteroids on weight change and previous studies have, in line with our results, also failed to show an association between weight gain and corticosteroid treatment in palliative cancer care [26, 32]. However, corticosteroids have been shown to increase appetite in the short term during a study period of 1–2 weeks [33, 34]. In a randomized, controlled study in patients with advanced cancer (*n* = 47), prednisolone treatment for 1 week improved appetite significantly [33]. Another randomized, double-blind controlled study in patients with advanced cancer (*n* = 84) showed a borderline significance of increased appetite after 2 weeks of dexamethasone treatment [34]. However, studies on the effect of corticosteroids on appetite in a longer perspective are lacking.

This study has several strengths and limitations that need to be addressed. To our knowledge, this is the first study performed to address the use of corticosteroids in patients with advanced pancreatic cancer evaluating the association with weight change and appetite during a study period of several months. This is also the first study to evaluate possible sex differences of corticosteroid treatment in this patient group. As study patients were recruited from across Sweden and represented a range of socioeconomic backgrounds and patients at different stages in the palliative disease trajectory, it enhances the likelihood that the cohort is representative of the broader population of patients with pancreatic cancer. A limitation is the attrition due to death during follow-up, which is unavoidable and a known challenge in all clinical studies performed in the palliative care setting [35]. Secondly, as this is an observational, post hoc study in a previously performed trial with another aim, there is a high risk of confounding-by-indication, and residual as well as unmeasured confounding cannot be ruled out. Since our analyses are based on observational data, we are unable to address the causality of the associations observed. Further, the study participants represent a selected cohort of patients who originally agreed to participate in the MISTRAL trial. Patients willing to participate may differ from the general cancer patient population within palliative care and, therefore, may not be representative of the broader palliative population. It is also likely that patients receiving corticosteroids may have had a higher risk of weight loss than those who were not prescribed corticosteroids, and that is why they received the treatment. Although there was a greater weight loss in the corticosteroid group also after adjustment for performance status (ECOG), the weight loss might have been even greater without corticosteroids. Thus, the association between corticosteroid therapy and weight change is still unclear. A final limitation is that appetite was measured by just one item in EORTC QLQ-C30. Although EORTC QLQ-C30 is a validated questionnaire, appetite could have been insufficiently captured, as a single item may not reflect the complex nature of appetite in this patient population.

## Conclusion

In conclusion, patients with pancreatic cancer receiving corticosteroid therapy experienced greater weight loss and reduced appetite in a long-term follow-up. However, when viewed in a broader clinical context, it is possible that corticosteroids were more frequently prescribed to patients in more advanced palliative stages with a higher symptom burden. Therefore, any observed associations between corticosteroid use and patient outcomes in this population should be interpreted cautiously. Since previously randomized, controlled studies on the effects of corticosteroids on appetite and weight change have only evaluated short-term use for a few weeks, there is a great need to perform prospective trials to evaluate the effect of corticosteroids in a long-term perspective in patients with advanced cancer.

## Data Availability

No datasets were generated or analysed during the current study.
